# An Unusual Presentation of an Amniotic Fluid Embolism: Fetal Bradycardia As the First Sign

**DOI:** 10.7759/cureus.67222

**Published:** 2024-08-19

**Authors:** Vicki Wang, Taizoon Q Dhoon, John Steller, Dominic Carusillo, Ramin Rahimian, Shermeen Vakharia, Joseph Rinehart

**Affiliations:** 1 Anesthesiology and Perioperative Medicine, UCI Health, Orange, USA; 2 Anesthesiology, UCI Health, Orange, USA; 3 Obstetrics and Gynecology, UCI Health, Orange, USA; 4 Anesthesiology and Perioperative Care, UCI Health, Orange, USA

**Keywords:** obstetric surgical procedures, cardiovascular complications, obstetric labor complications, pregnancy complications, embolism, disseminated intravascular coagulation, fetal bradycardia, maternal cardiac arrest, amniotic fluid embolism

## Abstract

Amniotic fluid embolism (AFE) is a potentially fatal maternal condition demanding awareness from obstetricians and anesthesiologists regarding its different manifestations. The typical presentation involves maternal respiratory distress, cardiovascular collapse, neurological changes, and coagulopathy followed by fetal distress.

This unusual case study emphasizes that fetal compromise may precede maternal decompensation as the initial sign of AFE. Fetal distress is a known symptom of AFE and is typically seen due to cardiorespiratory issues that lead to reduced uteroplacental perfusion, resulting in fetal hypoxia. In the case presented, fetal bradycardia occurred before any visible maternal symptoms, suggesting that fetal distress could be induced by factors independent of the mother's cardiopulmonary status.

A 34-year-old healthy G4P2012 at 41 weeks and 2 days gestation who was initially laboring on the floor was emergently taken to the operating room for a cesarean delivery due to fetal bradycardia. Around the time the fetus was delivered, the patient displayed seizure activity, followed by a complete loss of consciousness and cardiac arrest. The patient was intubated and underwent cardiopulmonary resuscitation and defibrillation, subsequently converting to a wide complex tachycardia. In the operating room, there was evidence of heavy vaginal bleeding, uterine atony, and a fulminant form of disseminated intravascular coagulopathy (DIC), which required aggressive management over the next four hours. After achieving hemodynamic stability, the patient was transferred to the surgical intensive care unit (SICU), extubated on day 3, and discharged home on day 8.

## Introduction

Amniotic fluid embolism (AFE) is a potentially lethal condition requiring obstetricians and anesthesiologists to be well-versed in both its common and uncommon manifestations to rapidly diagnose and initiate resuscitation. Despite advancements in resuscitation, the prognosis is often grim for both the infant and the mother. The exact incidence of AFE remains elusive, but it is estimated to occur in one in 8,000 to one in 80,000 deliveries. Older reports cite mortality rates as high as 60%, even with immediate and aggressive treatment. Maternal morbidity rates are also substantial, with only 15% of survivors remaining neurologically intact [1].

Typical symptoms at presentation include dyspnea, vaginal bleeding, and altered mental status [2,3]. These symptoms can quickly transition to cardiopulmonary collapse, seizures, hypotension, and disseminated intravascular coagulation (DIC) [2-4]. Non-reassuring fetal status is often a result of uteroplacental hypoperfusion and fetal hypoxemia/acidosis caused by the initial maternal insults above. Still, in rare circumstances, it can be the initial presenting sign [2]. The key to successful outcomes lies in quick suspicion and exclusion of competing diagnoses, timely and efficient escalation of management, and teamwork among a large multidisciplinary team.

The standard of care includes cardiopulmonary resuscitation (CPR), control of bleeding and reversal of coagulopathy, and delivery of the fetus [3,5]. Care often requires advanced cardiac life support, emergency airway management, and mechanical ventilation, the establishment of central venous access, rapid administration of intravenous fluids, vasopressor therapy, massive transfusion of blood products and factors, tocolytics after delivery, and administration of tranexamic acid [5-8]. Investigational therapies include C1 esterase inhibitors, other fibrinolytic agents, and the combination of atropine, ondansetron, and ketorolac as part of the A-OK protocol [6,7]. The patient provided written HIPAA authorization to publish this case report.

## Case presentation

A 34-year-old woman at 41 weeks and 2 days gestation presented for induction of labor given post-term gestation. Admissions labs were unremarkable (Table 1). She had a cervical ripening balloon placed and was started on 4 milli-units/min of oxytocin. A combined spinal epidural (CSE) procedure was performed for labor analgesia and was tolerated well. Ninety minutes following the CSE placement (approximately 22 hours after induction of labor) terminal bradycardia was noted in the setting of a previously Category 1 fetal heart rate tracing (FHRT). The obstetrics team immediately responded to the patient’s bedside. A fetal scalp electrode was placed which confirmed an FHR in the 70s. Standard uterine resuscitation measures were performed, including multiple position changes, oxytocin and epidural infusion discontinuation, IV fluid bolus, and terbutaline administration. The prolonged deceleration continued to the six-minute mark, at which point the patient was emergently taken to the OR for cesarean delivery if the FHRT was still non-reassuring.

**Table 1:** Hemogram and coagulation testing. (H) Data are abnormally high. (L): Data are abnormally low. Plt - Platelet, INR - international normalized ratio, PTT - partial thromboplastin time

Hemogram and coagulation test				
	Admission lab values	Initial resuscitation value(s)	Final resuscitation value(s)	Reference range
Hgb	12.6	10.3 (L)	9.3 (L)	11.5 – 15.0 g/dL
Plt	186	120 (L)	84 (L)	150 – 400 thousand/mcL
Prothrombin time	-	>120.0 (H)	11.0 (L)	12.0 – 14.2 seconds
INR	-	>12.00 (H)	0.80 (L)	0.89 – 1.11
PTT	-	>200.0 (H)	37.5 (H)	24.2 – 36.7 seconds
Fibrinogen	-	<30 (L)	213	155 – 439 mg/dL
D-dimer	-	-	>20,000 (H)	<500 ng/mL FEU

Upon entry to the OR, the patient was still alert and moved herself from the gurney to the operating room table. American Society of Anesthesiologists (ASA) standard monitors, including pulse oximetry (SpO2), were placed. Fetal bradycardia was still present. The patient was prepped in a sterile fashion, as the epidural catheter was activated with 10mL of chloroprocaine 3% in divided doses. The patient reported an adequate surgical level upon examination, and the surgeons made their incision. A viable male neonate was delivered within one minute of surgical incision (Apgar scores 5 and 9).

Around the time of the incision, the anesthesia team noticed a change in the patient’s mental status. Initially, this was attributed to inadequate neuraxial anesthesia and discomfort but rapidly progressed to agitation and seizure activity by the time the neonate was delivered (one minute). The patient then became bradycardic (50 bpm) with an undeterminable electrocardiogram (EKG) morphology, which raised suspicion of possible local anesthetic toxicity or high spinal.

Shortly thereafter, the patient rapidly transitioned to tachycardia (120 bpm) with another change in the appearance of the EKG morphology, followed by a complete loss of consciousness (LOC). Succinylcholine 100mg was administered followed by endotracheal intubation and activation of the emergency response team.

Following intubation, the patient’s vitals were 100% SpO2 saturation, non-invasive BP (NIBP) 109/53 mmHg, HR 80s, and temperature of 36° Celsius. Though the patient displayed seemingly normal vitals and had a palpable carotid pulse, the end-tidal CO2 (EtCO2) was below 10mmHg, the patient’s skin began to appear purple and mottled, and there was an absence of bleeding from the open hysterotomy which was highly unusual. Almost concurrently with the intubation, bedside ultrasound was used by another team member who had arrived to assist with the emergent cesarean section to perform a transthoracic echocardiogram (TTE), which demonstrated a virtually akinetic heart despite the reassuring heart rate, saturations, and blood pressure being shown on the monitor at the same time. CPR was initiated.

Return of spontaneous circulation (ROSC) was attained after one round of CPR. A Transesophageal echocardiogram (TEE) probe, a radial arterial catheter, and a central venous catheter were placed. Initial arterial blood gas demonstrated severe acidemia and anemia (Table 2). The patient was placed on norepinephrine and epinephrine infusions, and administered sodium bicarbonate, magnesium, calcium chloride, and tranexamic acid (TXA). Despite these interventions, the patient decompensated into ventricular fibrillation. The patient underwent a second round of CPR and defibrillation, and converted to a wide complex tachycardia.

**Table 2:** Point-of-care arterial blood gas (ABG) testing. (H): Data are abnormally high. (L): Data are abnormally low.

Point-of-care arterial blood gas			
	Initial ABG value(s)	Final ABG value(s)	Reference range
pH	7.17 (L)	7.35 (L)	7.38 – 7.42
PaCO2	42	42	36 – 42 mmHg
PaO2	276 (H)	181 (H)	80 – 104 mmHg
BE	-13	-2	-
HCO3	15 (L)	23	21 – 27 mmol/L
Hgb	8.8 (L)	9.2 (L)	11.5 – 15.0 g/dL

Over the next 30 minutes, the patient’s cardiac function slowly improved on TEE and inotropic support was gradually weaned off. Considering the patient's quick decline in health and exclusion of other likely causes, a diagnosis of AFE was suspected. The blood bank and transfusion medicine team were notified of the case. Ondansetron and Ketorolac were given intravenously as part of the A-OK regimen to mitigate the severe inflammatory response associated with AFE; atropine was withheld given the patient’s recent tachy-dysrhythmias.

Shortly after the surgical closure of the hysterotomy and abdominal wall, there was evidence of heavy vaginal bleeding (1,500mL) and uterine atony. Typically, DIC and postpartum hemorrhage (PPH) present immediately following delivery. In this case, hemorrhage started 30 minutes after delivery and surgical closure. The epinephrine and norepinephrine infusions were reinitiated. A Bakri balloon was placed and confirmed on ultrasound, and focused assessment with sonography for trauma (FAST) scans and uterine ultrasounds were performed periodically to monitor for ongoing bleeding. The massive transfusion protocol was initiated. An initial transfusion of four units of packed red blood cells (pRBC), four units of fresh frozen plasma (FFP), two units of cryoprecipitate (Cryo), and one unit of platelet (PLT) were transfused. Labs collected after this initial resuscitation revealed a severely deranged coagulation profile with an elevated INR and low fibrinogen. Rotational thromboelastometry (ROTEM) failed to clot for almost an hour (Table 3). Additionally, she continued to have anemia and thrombocytopenia (Table 1).

**Table 3:** ROTEM testing. (H): Data are abnormally high. (L): Data are abnormally low. EXTEM - extrinsically activated test, FIBTEM - fibrin-based extrinsically activated test

Rotational thromboelastometry (ROTEM)			
	Initial ROTEM	Final ROTEM	Reference range
EXTEM clotting time (CT)	3.327 (H)	44	43 - 82 sec
EXTEM clot formation time (CFT)	-	229 (H)	48 - 127 sec
EXTEM alpha angle	-	50 (L)	65 - 80 degree
EXTEM amplitude achieved 10 minutes after CT (A10)	-	40	mm
EXTEM amplitude achieved 20 minutes after CT (A20)	-	49 (L)	50 - 70 mm
EXTEM maximum clot firmness (MCF)	-	55	52 - 70 mm
EXTEM maximum lysis (ML)	-	0	<15%
FIBTEM CT	3.594 (H)	36	sec
FIBTEM alpha angle	-	45	degree
FIBTEM amplitude achieved 10 minutes after CT (A10)	-	9	mm
FIBTEM amplitude achieved 20 minutes after CT (A20)	-	9	7 - 24 mm
FIBTEM MCF	-	10	7 - 24 mm
FIBTEM ML	-	0	%

Following the initial resuscitation, the patient entered fulminant DIC. During the case, the patient received a total of 9 units of pRBCs, 13 units of FFP, three units of PLT, and 8 units of Cryo, as well as 2.3 liters of intravenous crystalloids. The patient also received recombinant factor VII and Vitamin K after extensive discussion with the blood bank’s pathologist. The estimated blood loss for the case was 3.1 liters. Arterial blood gas at the case's conclusion showed gross improvement in her acid-base physiology with only mild acidemia (Table 2). Formal laboratory studies were collected and sent at that time, which showed persistent anemia, mild thrombocytopenia, decreased PT/INR, elevated D-Dimer, and normal fibrinogen levels. ROTEM testing was again performed which revealed normal fibrin-based extrinsically activated test (FIBTEM) and prolonged extrinsically activated test (EXTEM) clot formation time (Tables 1, 3).

**Figure 1:**
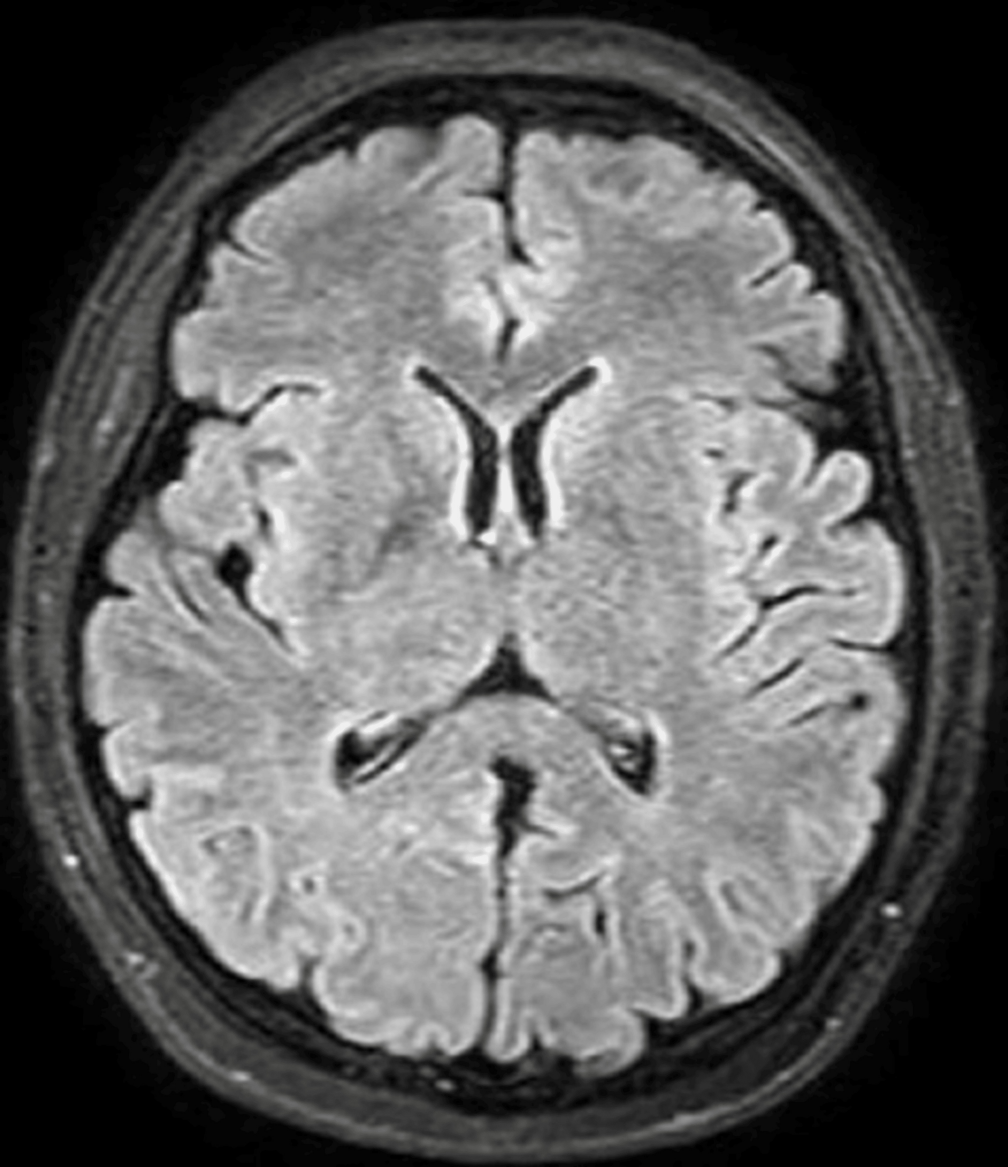
Magnetic resonance imaging (MRI) of the brain, taken on the post-operative day (POD) 1, revealed no signs of acute abnormalities.

The patient was transferred to the SICU in hemodynamically stable condition and weaned off inotropic support five hours after surgical incision. The patient remained in the ICU for three days. On the postoperative day (POD) 1, the hemoglobin remained stable at 7.9mg/dL and ROTEM indicated no further need for transfusion. Despite sedation being weaned overnight, the patient was minimally responsive initially during morning rounds. Computed tomography (CT), CT angiography, and magnetic resonance imaging (MRI) were performed which indicated no acute bleeds, parenchymal changes, or vascular abnormalities (Figure 1). Neurology consultation concluded that the persistent level of altered consciousness was likely due to generalized encephalopathy related to the AFE and/or cerebral hypoperfusion around the time of cardiac arrest, rather than focal stroke.

By POD 3, the patient’s neurological status had improved, and she was successfully extubated. At the time of discharge to home on POD 8, the patient did exhibit a loss of memory around the time of her delivery and immediate post-operative course; however, her cognition had returned to its baseline level, and she was fully capable of self-care. She was started on oral ferrous sulfate and discharged for routine postpartum care. Both mother and baby continued to do well at the time of their six-month follow-up visit.

## Discussion

AFE is a rare complication of pregnancy with a mortality rate of 20% to 60% [5]. It is hypothesized that contents of amniotic fluid that enter the mother’s systemic circulation can trigger an anaphylactoid response, cytokine storm, and coagulation storm. Previous theories regarding the pathophysiology of AFE focused on the role of amniotic fluid's physical components obstructing maternal blood vessels [6]. However, recent theories propose that the maternal immune system is activated in response to fetal cells, antigenic material, or bacterial antigens that infiltrate the maternal systemic circulation through a rupture in the maternal-fetal interface. This can initiate an unusual humoral and immunological response, leading to the release of vasoactive and procoagulant substances [7,8]. Such a response can cause cell and tissue damage, systemic vasoplegia, capillary leakage, pulmonary vasoconstriction, bronchospasm, DIC, hemorrhagic or distributive shock, and cardiovascular collapse. Consequently, an AFE may manifest as a mix of several conditions including a cytokine storm, anaphylactoid syndrome, immune storm, and coagulation storms [5].

Patients with AFE may exhibit a constellation of symptoms including neurological symptoms (agitation, a feeling of impending doom, altered mental status, seizures, or encephalopathy); cardiovascular symptoms (hypotension, acute pulmonary hypertension, or right or left heart failure), DIC, uterine atony, and fetal bradycardia. AFE is typically described as sudden hypotension or cardiac arrest, respiratory distress or hypoxia, coagulopathy, or lack of fever, and typically occurs during labor, cesarean delivery, dilation, and evacuation, or within 30 minutes postpartum.

Management requires a rapid and accurate diagnosis as patients with AFE can quickly decompensate; this can be challenging as AFE is a diagnosis of exclusion. The typical presentation of AFE follows a timeline of cardiac and respiratory symptoms appearing first in the mother with most cases occurring during labor. Classical findings include sudden cardiovascular collapse, seizures, and respiratory difficulty. A triad of hypotension, coagulopathy, and hypoxia is often described. However, symptoms can overlap with more common adverse events associated with pregnancy. Atypical presentation of AFE occurs in 25% of cases making diagnosis more complex [5].

In the current case, it was clear that an insidious process was quickly unfolding. Several possibilities were considered once the patient's mother started showing signs of distress. These included insufficient neuraxial anesthesia due to the patient's initial restlessness and discomfort; local anesthetic systemic toxicity (LAST), high spinal, or seizure, as indicated by the LOC and irregular heart rhythm; and lastly, AFE during intubation when a markedly low ETCO2, an akinetic heart on TTE and almost total cardiovascular collapse, and DIC. AFE was determined to be the most likely etiology given the constellation of symptoms.

Fetal distress is a recognized symptom of AFE, but it is typically seen due to cardiorespiratory compromise leading to uteroplacental perfusion and thus fetal hypoxia. In this instance, fetal bradycardia preceded any overt signs from the mother, who was breathing and mentating normally, and capable of moving herself to the operating table six minutes into the insult. Although the patient eventually exhibited seizure, cardiac arrest, and coagulopathy, her vital signs including blood pressure, O2 saturation, and neurologic function remained stable until she was fully decompensated. By this point, the baby had already been delivered. A similar sequence of events, where fetal bradycardia occurred without maternal cardiovascular collapse was the initial symptom of AFE, has been documented in a previous case report [9]. 

These cases support the idea that fetal distress may be triggered by mechanisms independent of the mother's cardiopulmonary status and instead linked to the same inciting factor that allowed amniotic fluid to enter the maternal circulation in the first place. We posit that an insult to the placental/maternal vascular interface could obstruct oxygen transfer from the maternal to fetal side, potentially leading to immediate fetal bradycardia and simultaneously triggering a maternal inflammatory, anaphylactoid, and/or thrombotic response. 

The pathological analysis of the placenta in this case revealed a 506-gram singleton placenta, standard umbilical cord with a three-vessel insertion, and typical fetal membranes. Importantly, there were signs of increased patchy intervillous fibrin deposition and an intraplacental thrombus measuring 3.2 cm. These observations support the theory that the intramural thrombus could have resulted in a placental infarct or might represent a type of placental abruption, resulting in enhanced vascular permeability and a heightened risk of an AFE.

In this case, the favorable outcomes for both the mother and the baby can be partially attributed to the initial presentation of fetal bradycardia, and the timely and effective management of the multi-disciplinary team of obstetricians, anesthesiologists, nursing staff, blood bank, and the laboratory. Infant survival rates are usually 70% and correlate with the time between delivery and maternal distress [5]. The prolonged fetal bradycardia influenced the early decision to move to the operating room where optimized space and resourcing for managing her seizure, LOC, cardiac arrest, and DIC were available.

## Conclusions

AFE is a life-threatening event, necessitating obstetricians and anesthesiologists to be well-versed in the signs and symptoms of both typical and atypical presentations of AFE. This specific case highlighted the unique occurrence of fetal compromise as the initial indicator of AFE. Favorable outcomes are reliant on a high index of suspicion, prompt diagnosis, and effective management. Additionally, close communication and partnership between obstetricians, anesthesiologists, neonatologists, blood banks, transfusion medicine specialists, intensivists, and other healthcare providers increase the likelihood of a favorable outcome.
